# Sarcomere dynamics revealed by a myofilament integrated FRET-based biosensor in live skeletal muscle fibers

**DOI:** 10.1038/s41598-022-21425-8

**Published:** 2022-10-27

**Authors:** Ashley A. Martin, Brian R. Thompson, Jonathan P. Davis, Hluechy Vang, Dongwoo Hahn, Joseph M. Metzger

**Affiliations:** 1 Department of Integrative Biology and Physiology, University of Minnesota Medical School, 6-125 Jackson Hall, 321 Church Street SE, Minneapolis, MN 55455 USA; 2 Department of Physiology and Cell Biology, Ohio State University, Columbus, OH USA

**Keywords:** Physiology, Skeletal muscle

## Abstract

The sarcomere is the functional unit of skeletal muscle, essential for proper contraction. Numerous acquired and inherited myopathies impact sarcomere function causing clinically significant disease. Mechanistic investigations of sarcomere activation have been challenging to undertake in the context of intact, live skeletal muscle fibers during real time physiological twitch contractions. Here, a skeletal muscle specific, intramolecular FRET-based biosensor was designed and engineered into fast skeletal muscle troponin C (TnC) to investigate the dynamics of sarcomere activation. In transgenic animals, the TnC biosensor incorporated into the skeletal muscle fiber sarcomeres by stoichiometric replacement of endogenous TnC and did not alter normal skeletal muscle contractile form or function. In intact single adult skeletal muscle fibers, real time twitch contractile data showed the TnC biosensor transient preceding the peak amplitude of contraction. Importantly, under physiological temperatures, inactivation of the TnC biosensor transient decayed significantly more slowly than the Ca^2+^ transient and contraction. The uncoupling of the TnC biosensor transient from the Ca^2+^ transient indicates the biosensor is not functioning as a Ca^2+^ transient reporter, but rather reports dynamic sarcomere activation/ inactivation that, in turn, is due to the ensemble effects of multiple activating ligands within the myofilaments. Together, these findings provide the foundation for implementing this new biosensor in future physiological studies investigating the mechanism of activation of the skeletal muscle sarcomere in health and disease.

## Introduction

The functional unit of skeletal muscle, the sarcomere, consists of a highly organized repeating pattern of contractile and regulatory proteins aligned into thick and thin filaments^1–3^. Here, the thick filament is composed of the molecular motor protein myosin. Myosin is able to interact with both actin and ATP, the hydrolysis of which is necessary for contraction of the sarcomere^1^. The thin filament consists of actin monomers that polymerize to form a tight helix. Essential regulators of sarcomere activation are located in the thin filament. The proteins tropomyosin (Tm) and the heterotrimeric troponin complex, consisting of troponin C (TnC), troponin I (TnI), and troponin T (TnT), work in concert, along with calcium, to regulate thin filament activation and muscle contraction^2,3^. Within the troponin complex, TnC is the calcium-binding subunit. In physiological conditions, electrical stimulation of the muscle leads to an increase in intracellular calcium, released from the sarcoplasmic reticulum (SR)^4^. Ca^2+^ binds to TnC causing the protein to undergo a conformational change that in turn initiates troponin complex and tropomyosin structural changes that are transmitted cooperatively along the entire thin filament. Through these interactions the myofilament becomes activated and force can be generated^2,3^. Central to this signaling mechanism is TnC, which serves as a nexus point for detecting and integrating thin and thick myofilament-based activating ligands^1^.

Currently, an understanding of sarcomere activation, including inter-myofilament signaling, has come into focus through the findings of numerous foundational studies, including biophysical studies using permeabilized or reconstituted systems^1,5^. These studies have established the role of the troponin complex in regulating thin filament activation through the binding of calcium to TnC^6–9^. This further includes studies on the function of thick filament in muscle contraction activation^5^, shown via myosin cross-bridge attachment to the thin filament and the resultant conformational changes of TnC^10–12^. More recently, studies using X-ray diffraction in both intact and permeabilized muscle have furthered the understanding of the impact of disease on the structure of the sarcomere^13,14^. While these works have been highly informative, they also have some significant limitations as the full physiologic structure of the muscle is often deficient in these investigations, including lack of intact excitation–contraction coupling processes and the intracellular physiological milieu.

To date, the ability to interrogate mechanisms of physiological sarcomere activation in an intact skeletal muscle system has been technically challenging to achieve. To address this experimental gap, we present here the design and implementation of a new skeletal muscle specific sarcomere activation biosensor system. Utilizing the distance-dependent nature of Forster/fluorescence Resonance Energy Transfer (FRET)^15^, this myofilament incorporated sarcomere activation biosensor was designed and engineered with the goal to provide real-time data on skeletal muscle fiber sarcomere activation dynamics. The FRET-based biosensor was constructed using full length human fast skeletal TnC (fsTnC) fused with the green fluorescent protein Clover (FRET donor) on the N-terminal and the red fluorescent protein mRuby2 (FRET acceptor) on the C-terminal. This biosensor construct was expressed in a transgenic mouse system and incorporated into myofilaments via stoichiometric replacement of endogenous TnC. In design, we considered that the ideal FRET pair should have a high level of fluorescent brightness and low sensitivity to environmental changes, and the expression of the FRET pair should not negatively impact the function, localization, or structure of protein they are fused with^16^. Based on these important features, we selected Clover as the FRET donor molecule and mRuby2 as the acceptor FRET molecule. At the time of this study, Clover and mRuby2 had the highest level of fluorescent brightness, largest FRET dynamic range, were the most photostable, and had the lowest levels of phototoxicity^17,18^.

In this work, we leveraged the global TnC conformational change that occurs during each twitch contraction as the integrating signaling event denoting sarcomere activation. As previous in vitro work has demonstrated the ability of FRET-based probes on TnC to track changes in TnC activation in the steady state^19^, this study sought to evaluate if this approach could translate physiologically to the intact skeletal muscle fiber. The hypothesis tested is the skeletal muscle biosensor will allow for the monitoring of sarcomere activation, through conformational changes in TnC, in real time in an intact system. Data show that with physiologically-relevant temporal resolution, the highly orchestrated sequence of events underscoring the kinetics of sarcomere activation and inactivation during a skeletal fiber twitch contraction can be observed.

## Methods

### Animal studies and Ethics

All animal experiments were conducted in agreement with the international ARRIVE guidelines^20^. The procedures and experiments used in this study were approved under guidelines of the University of Minnesota Institutional Animal Care and Use Committee (IACUC). Animal euthanasia was performed using cervical dislocation in isoflurane anesthetized animals in accordance with the American Veterinary Medical Association (AVMA) guidelines^21^. Skeletal muscles were isolated from adult C57/Bl6 mice (Jackson Labs) or adult fsTnC biosensor transgenic mice of both sexes.

### Biosensor design

**Figure 1:**
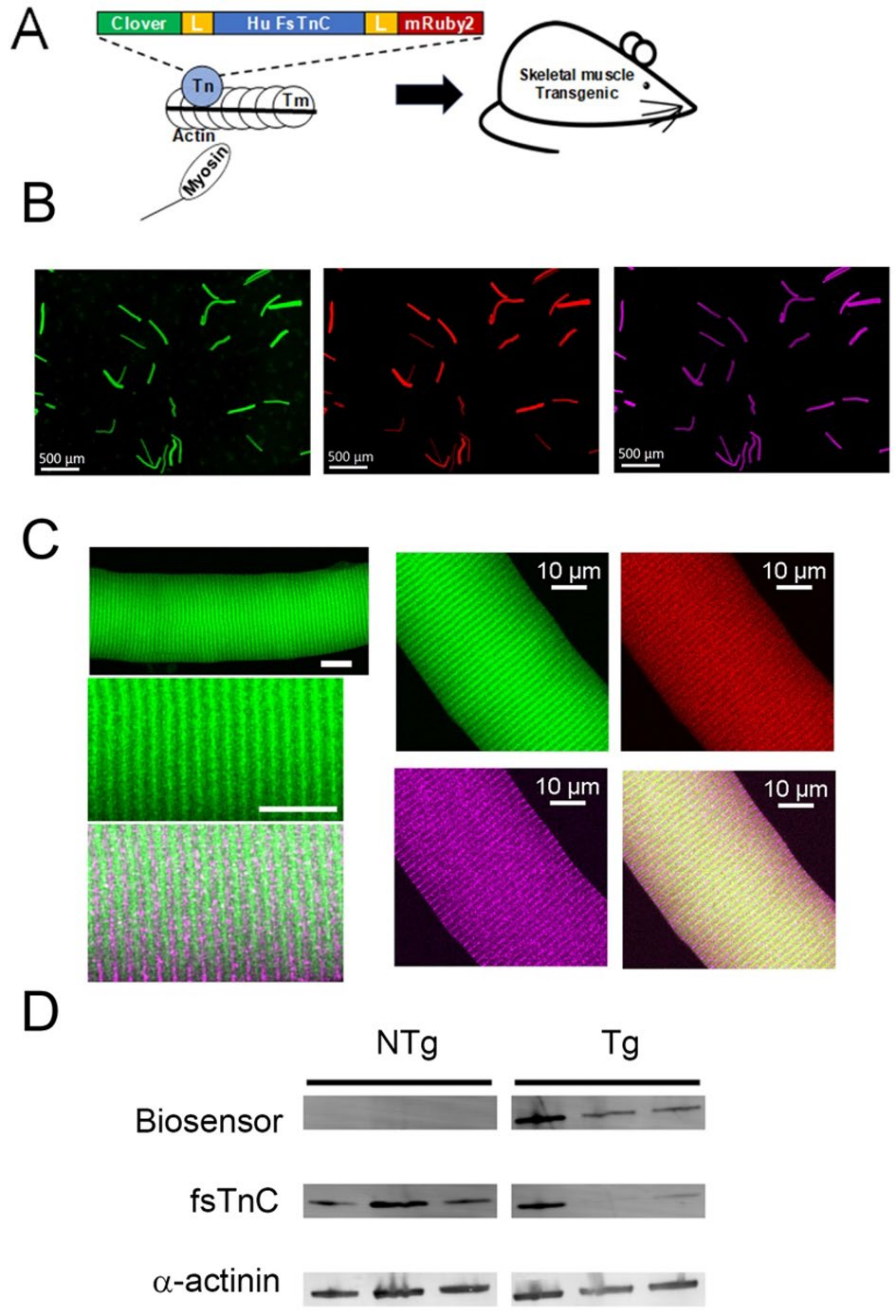
TnC biosensor transgene construction, localization, and expression. (**A**) The human fast skeletal muscle troponin C (h-fsTnC) cDNA was engineered and flanked on the 5’-end by a 19 amino acid flexible linker and the green fluorescent protein Clover and on the 3’-end by an 8 amino acid flexible linker and the red fluorescent protein mRuby2. (**B**) Low magnification immunofluorescence images of isolated FDB fibers demonstrating Clover (green) and mRuby2 (red) expression and α-actinin staining (purple) (scale bar = 500 μm). (**C**) High magnification immunofluorescence images of isolated FDB fibers. Left panel contains confocal images which show myofilament localization of the TnC biosensor (Clover-green, α-actinin – purple) (Top scale bar = 20 μm, bottom scale bar = 10 μm). Right panel images of same FDB fibers demonstrating co-localization of Clover (green) and mRuby2 (red) resulting in yellow (lower right panel) in correct orientation between Z-lines show by α-actinin staining (purple). (**D**) Western blot of fsTnC expression probed with anti-fsTnC antibody in transgenic EDL myofibers shows stoichiometric replacement of endogenous fsTnC (18 kDa) by the higher molecular weight biosensor fsTnC (72 kDa). The blot was also probed with an antibody for α-actinin (103 kDa) as a loading control. For clarity, the blot has been spliced to remove intervening non-specific bands. The full, labelled, blot is provided in the supplement (Supplementary Figs.1, 2).

An intramolecular Clover/mRuby2 FRET pair was engineered onto human fast skeletal troponin C (Fig. 1A). Here, the 480 bp full-length human fast skeletal troponin C cDNA (h-fsTnC), minus the starting methionine, was synthesized de novo and flanked on the 5′ end by a 19 amino acid flexible linker peptide enriched in leucine, glycine, serine, and alanine, and flanked on the 3′ end by an 8 amino acid flexible linker enriched in leucine and alanine. Linker design was based, in part, on previous works showing a range of linker lengths in the construction of a cardiac TnC CFP/YFP construct, importantly these linkers do not affect the Ca^2+^/Mg^2+^ binding characteristics of the isolated TnC^19^. A *Pas*I restriction site at the 5′ end and an *Eco*RV restriction cite at the 3′ end allowed for the insertion of the 19 amino acid flexible linker. The 8 amino acid flexible linker was inserted between a 5′ *Pfo*I restriction site and a 3′ *Bcl*I restriction site. The 5′ end of the 19 amino acid flexible linker is the site of the 684 bp cDNA sequence of the green fluorescent protein Clover. The Clover cDNA was inserted between a *Sal*I restriction site on the 5′ end and the previously mentioned *Pas*I restriction site on the 3′ end. The 3′ end of the 8 amino acid flexible linker is the location of the 714 bp cDNA sequence of the red fluorescent protein mRuby2. The mRuby2 cDNA is inserted between the previously mentioned *Bcl*I restriction site on the 5′ end and an *Fse*I restriction site on the 3′ end.

The h-fsTnC biosensor gene construct was inserted in a DNA vector downstream of a human skeletal muscle actin α1 (ACTA1) promoter element for skeletal muscle specific expression in the mammalian system. Due to the h-fsTnC being highly conserved between mammalian species, this construct can be used in the comparison to results from previous studies which also used human TnC^19,22^. This ACTA1 h-fsTnC biosensor vector was inserted by pronuclear injection into donor egg cells and implanted in C57/Bl6 murine surrogate mothers. Live pups were genotyped using a PCR forward primer sequence located in the ACTA1 promoter: 5′-GCCGAGAGTAGCAGTTGTAGC-3′, and a PCR reverse primer located in the Clover sequence: 5′-TAGCCAAATGTGGTCACGAGG-3′ with an annealing temperature of 60 °C. Of the live pups, 5 founders carrying the transgene were identified and the line with greatest breeding vigor was selected for further validation.

The FRET pair of Clover and mRuby2 was selected because, at the time of this study design, this fluorophore pair was superior to previously studied FRET pairs in terms of fluorescence intensity, brightness, and photostability. This pair also had the highest Forester radius of any currently described ratiometric FRET pair^17–19^. Clover (excitation max/emission max = 505/515 nm) and mRuby2 (excitation max/emission max = 559/600 nm) were used due to the high extinction coefficient and extensive excitation overlap of mRuby2 with Clover emission, which yields a R_0_ of 6.3 nm, along with high quantum yield, large Stokes shift, fast maturation, and high photostability^17,18^, making mRuby2 and Clover outstanding FRET partners. Additionally, when compared to previously used CFP/YFP pairs, this mRuby2/Clover pair has an exceptional signal-to-noise ratio for fluorescence levels with significantly less phototoxicity leading to superior FRET imaging^17,18^.

### Skeletal muscle morphology, fiber typing, Western blotting, and immunofluorescence

After first obtaining body weights, 4–6 month old transgenic (Tg) and non-transgenic (NTg) mice of both sexes were euthanized by cervical dislocation. Tibialis anterior (TA), gastrocnemius, extensor digitorum longus (EDL), and soleus (SOL) muscles were excised, making sure to remove excess tendons, and wet weight was measured, these measurements were then normalized to corresponding tibia lengths.

Fiber typing was done on isolated whole EDL and SOL muscle, which were removed as described above. Muscles were embedded in O.C.T. compound (Tissue-Tekk; Sakura) and then flash frozen in liquid nitrogen cooled isopentane. Once embedded, samples were stored at − 80 °C until sectioned. Sections were taken from the mid-belly section of the tissue and were cut in 10 μm thick cryosections using a cryostat (Leica) which was held at − 22 °C. The sections were subsequently used for immunofluorescence analysis of myosin heavy chain (MHC) expression. At room temperature, sections were first air dried for 10 min and then blocked for one hour in 5% bovine serum albumin in phosphate buffer saline (PBS). They were then incubated with primary antibody (fast skeletal MHC: Abcam MY-32, slow skeletal MHC: Accurate Chemical and Scientific Corp. MEDCLA67-1) at a 1:1000 dilution in blocking solution for 120 min. Following incubation, the sections were washed three times for five minutes each using PBS. The sections were then incubated with secondary antibody (Alex Fluor 488; Thermo Fisher) at a 1:500 dilution in blocking solution for 1 h. Following secondary incubation, the sections were again washed 3 times for five minutes each using PBS. Sections were then mounted using ProLong Diamond antifade mounting medium (ThermoFisher) and allowed to set at room temperature overnight in the dark. Images were collected using a Nikon AZ100 Motorized Fluorescence Microscope (Nikon) at 10x. For fiber type analysis, all fibers within the entire muscle/cross-section were characterized.

For Western blots, a myofibrillar isolation was first done as previously described^23^. Briefly EDL muscles were isolated from non-transgenic and transgenic animals and homogenized (PowerGen 125) in 0.3 M sucrose with 10 mM imidazole on ice. The homogenized tissue was then centrifuged at 13,500 rpm for 20 min at 4 °C. The supernatant was removed, and the pellets were resuspended in standard buffer solution (60 mM KCl, 30 mM imidazole, 2 mM MgCl_2_, pH 7). Then the suspension was centrifuged at 2800 rpm for 15 min at 4 °C. The above steps were repeated four more times and the resulting myofibrils were resuspended in the standard buffer solution containing 2 mM EGTA. This suspension was spun down at 2800 rpm for 15 min. The EGTA-treated pellet was resuspended in standard buffer solution containing 1% Triton X-100 and again spun down at 2800 rpm for 15 min, this step was repeated once. The final pellet was washed 3–4 times with standard buffer solution and, once washed, resuspended in standard buffer solution, and stored at − 80 °C. The primary antibodies used were for fast skeletal troponin C (E-7, Santa Cruz, 1:1800) and α-actinin (1:2000, NBP1-3257) and the secondary antibodies were the IRDyes Alexa Fluor 680 and Alexa Fluor 800 (ThermoFisher, 1:10,000). The Western blot signal was visualized and quantified using the Odyssey system (Licor). To quantify replacement of endogenous TnC with the biosensor, the intensity of the biosensor band was divided by total intensity of the endogenous band and the biosensor band combined. These calculations were done only within the same lane as they are single sample comparisons.

Localization of the biosensor was visualized by using isolated single myofibers from FDB muscles and plated onto laminin-coated coverslips. These coverslips were treated with 4% paraformaldehyde for approximately 20 min to fix the myofibers followed by two rounds of washes using phosphate buffer saline (PBS). The myofibers were blocked using a 5% bovine serum albumin solution with 0.5% Triton X-100 for approximately 1 h. The coverslips were then incubated with primary antibody [α-actinin 1:500 (NBP1-32578)] diluted in blocking solution for one hour. Next three 5 mins washes with PBS were done and the secondary antibody [Alexa Fluor 647 1:500 (ThermoFisher)], diluted in blocking solution, was incubated for one hour. Again, three rounds of five-minute washes of PBS were completed and coverslips were mounted on microscope slides using ProLong Diamond mounting medium. Slides were allowed to cure for 24 h at room temperature in the dark before imaging. Myofibers were imaged using a Nikon C2 confocal microscope with a 60 × oil-immersion objective. Images were obtained using in-line scanning mode with 488 nm, 561 nm, and 640 nm lasers.

### TnC protein isolation and Ca^2+^ dissociation rates

Human fast skeletal troponin C with and without the intramolecular Clover/mRuby2 FRET pair were expressed and isolated using BacPower bacterial protein expression system (GenScript). The expression and isolation protocol involves the addition of a 6-His tag on the C-terminal end of the protein product which remained throughout the following studies.

All kinetics measurements were carried out at 20 °C using an Applied Photophysics Ltd. (Leatherhead, UK) model SX.18MV stopped-flow apparatus, with a dead time of ∼1.4 ms. The apparent and actual rates of Ca^2+^ dissociation were measured from the fast skeletal control TnC in the presence and absence of the C-terminal, regulatory domain of fast skeletal TnI (residues 96–148) following a change in Tyr and quin-2 fluorescence, respectively. Quin-2 fluorescence was excited at 330 nm and monitored using a 510 nm broad filter (Oriel, Stratford, CT), whereas Tyr fluorescence was excited at 275 nm and monitored using UG1 filter (Oriel, Stratford, CT), as previously reported^24,25^. The apparent rates of Ca^2+^ dissociation from the fast skeletal FRET TnC were measured by following a change in mRuby2 or Clover fluorescence with an excitation wavelength of 505 nm. Clover fluorescence was monitored using a 530 nm narrow filter (Oriel, Stratford, CT), whereas mRuby2 fluorescence was monitored using a 600 nm narrow filter (Oriel, Stratford, CT). 10 mM EGTA in stopped-flow buffer (10 mM MOPS, 150 mM KCl, at pH 7.0) was used to remove Ca^2+^ (200 μM) from the various TnCs (3 uM) in the absence or presence of fast skeletal TnI ((residues 96–148) 9uM) also in the stopped-flow buffer. Tween-80 (0.02 percent) was added to the buffers with the FRET TnC to minimize non-specific interactions of the proteins. Both the control data sets (200uM Ca^2+^ instead of EGTA or 10 mM EGTA added to the proteins) resulted in a small and consistent time-dependent decrease in fluorescence that was subtracted from the datasets using the Ca-vs-Ca control shots. Data traces were fit using a program (by P.J. King, Applied Photophysics Ltd.) that utilizes the nonlinear Levenberg–Marquardt algorithm. Each *k*_off_ represents an average of at least seven separate experiments ± standard error, each averaging at least three individual shots fit with a single or double exponential equation.

### Steady-state force and TnC biosensor signal in permeabilized extensor digitorum longus muscle

EDL muscles were isolated from 8 to 12 week old mice of both sexes which had been euthanized by cervical dislocation. Once muscles were dissected they were kept whole for NTg vs Tg force studies or cut in half length-wise for force vs ratio studies. Then platinum foil clips in the shape of an omega (Ω) were constructed and used to attach the tendons of the muscle to the apparatus using 7–0 silk sutures. EDL muscles were then incubated in skinning solution [100 mM KCl, 10 mM Imidazole, 2 mM EDTA, 1 mM MgCl_2_ (Fluka Analytical), 4 mM ATP, 50% glycerol, protease inhibitor (Pierce Protease Inhibitor Tablets A32963), 30 mM BDM, 1% *w/v* Triton X-100] at 4 °C for approximately 48 h. After incubation, EDL muscles were individually placed in 1.5 ml microcentrifuge tubes filled with approximately 1 ml of storage solution [100 mM KCl, 10 mM Imidazole, 2 mM EDTA, 1 mM MgCl2 (Fluka Analytical), 4 mM ATP, 50% glycerol, protease inhibitor (Pierce Protease Inhibitor Tablets A32963)] and stored at − 20 °C for no more than 14 days. At the time of experiment, an EDL muscle was mounted by the platinum clips in a flow chamber (IonOptix) filled with relaxing solution [100 mM KCl, 10 mM Imidazole, 2 mM EDTA, 1 mM MgCl_2_ (Fluka Analytical), 4 mM ATP] between hooks connected to a length-controlled micromanipulator and a force transducer (MyoTronic). The temperature of the chamber was set by ambient room temperature, approximately 22 °C. The strip was stretched to an optimum physiological sarcomere length of 2.4–2.5 μm as detected by a MyoCam (Ionoptix). Skinned EDL muscles were activated using solutions of pCa 9, pCa 7, pCa 6.5, pCa 6, pCa 5.7, pCa 5, and pCa 4. To evaluate calcium driven tension versus ratio changes, muscles from transgenic animals expressing the biosensor were used and changes in developed tension and fluorescence intensity were simultaneously recorded. For evaluating tension changes between transgenic and non-transgenic EDL muscles only changes in developed tension were recorded. Both sets of experiments were done using the Ionoptix Calcium and Contractility system. Recorded forces were normalized to pCa 4 values for each individual preparation and recorded fluorescence intensities were normalized to pCa 6. These values were then fit to the Hill equation^26^:

$$F=\frac{1}{1+({10}^{\left(x-pCa50\right)}{)}^{nHill}}$$

with pCa50 being the calcium concentration at half activation and nHill representing the Hill coefficient, using non-linear least squares regression (Graphpad PRISM version 6.0; GraphPad Software Inc.).

### Flexor digitorum brevis single skeletal muscle fibers

Eight to twelve week old mice of both sexes were euthanized by cervical dislocation. The paws were removed and submerged in Krebs–Henseleit Buffer (KHB) supplemented with 40 mM BDM. Intact flexor digitorum brevis (FDB) muscles were carefully dissected from each paw. The isolated muscles were then incubated in M199 media (Gibco cat# 31100-035) with 10% fetal bovine serum and 2 mg/ml crudely purified collagenase for 3 h at 37 °C. Following incubation, the muscles were gently mechanically triturated in fresh M199 media to release individual myofibers. The dissociated myofibers were allowed to settle in M199 media for 15 min at 37 °C. Myofibers were then plated on laminin-coated (ThermoFisher cat# 23017015) glass coverslips in M199 media containing 5% fetal bovine serum and incubated for 1 h at 37 °C with 5% CO_2_. After the one-hour incubation, the media was changed to serum free M199 media and the myofibers were used for experiments within 5 h.

### Simultaneous contractility and TnC FRET measurements in isolated FDB muscle fibers

For real-time, simultaneous sarcomere length and TnC biosensor FRET fluorescence measurements, a modified Ionoptix Calcium and Contractility system was used, similar to as previously described in a cardiac biosensor system^22^. Sarcomere length changes were detected at a 240 Hz sampling rate and fluorescence measurements were detected at 1000 Hz sampling rate using two photomultiplier tubes. Measurements were made at room temperature, approximately 22 °C, and a stimulation frequency of 0.2 Hz, unless otherwise specified. Room temperature was selected as it is standard in the field and should not have any deleterious effect on the studies^27^. As it has recently been shown that temperature may impact the function of the thick filament^28,29^ some experiments were also carried out at 37 °C. The pacing was chosen to prevent muscle fatigue and rundown through data collection.

### Calcium transient measurements

For experiments examining calcium fluorescence in intact, isolated FDB fibers, fibers were preloaded for 10 min in M199 solution containing 1 μM Fura-2 AM (ThermoFisher #F1221) and a 15 min de-esterification was allowed. For the fluorescence measurements the 360/380 nm ratio was recorded. In each fiber several contractions were measured, averaged, and analyzed using the IonWizard software. Background subtraction was done by collecting fluorescence from an area away from any myofibers and subtracting that value from those recorded during contractions. Measurement parameters were as described above.

### Analysis and statistics

For sarcomere length, biosensor transient, and calcium transient analysis curve fitting was performed on unfiltered, averaged single myofiber transients (IonWizard 6.5; IonOptix) using a truncated Taylor series expansion. Additional details regarding the fitting methodology are available from IonOptix. To address changes in the fluorophore concentration during FDB myofiber isometric twitches, ratiometric analyses were performed. This analysis takes advantages of the intramolecular FRET pairing of the biosensor and its 1:1 fluorophore stoichiometry. The equation used to calculate the ratio is as follows:

$${Ratio}_{Fluorescence }= \frac{{I}_{mRuby2}}{{I}_{Clover}}$$

where *I* refers to the fluorophore intensity counted using the PMT. The intensity of the mRuby2 fluorophore signal is ratioed against the intensity of the Clover fluorophore signal. Thus, increases in mRuby2 intensity greater than a proportional increase in Clover intensity are due to an increase in the level of FRET between the two fluorophores^30^. Unbiased experimental design was used, including blinding of samples when possible, and the use of both male and female mice. Animals were used between 8 and 16 weeks old, and sample sizes have been determined by a Power analysis informed by previous experience in the laboratory with respect to biological variability due to sex, rigor, and reproducibility^22^. Mean ± S.E.M. data are presented in summary figures and two-tailed unpaired *t*-tests were performed (Graphpad PRISM version 6.0; GraphPad Software Inc.). Sample sizes were established based on Power analyses, as informed by previous laboratory experience with respect to biological variability. *P* values < 0.05 were considered statistically significant and indicated by asterisks in figures.

## Results

### TnC biosensor integrates into the sarcomere and functions similar to wild type TnC

The human fast skeletal muscle TnC (fsTnC) biosensor was conceptualized based on the principles of FRET (Forster/fluorescence Resonance Energy Transfer) to provide a powerful tool to visualize distance-based protein conformation changes with highly accurate spatio-temporal resolution. The FRET pair includes a donor and complimentary acceptor fluorophore, with the donor able to non-radiatively transfer excitation energy to the nearby acceptor fluorophore. This allows the acceptor fluorophore to emit its fluorescence wavelength^31^.

The fsTnC biosensor is an intramolecular FRET pair with full-length human TnC fused with the green fluorescent protein Clover, as the donor, on the N-terminal end and the red fluorescent protein mRuby2, as the acceptor, on the C-terminal end (details in “Methods”). Skeletal muscle directed fsTnC biosensor mouse lines were then established (“Methods”, Fig. 1A). Localization of the TnC biosensor was determined in single muscle fibers by immunofluorescence, which demonstrated that in isolated flexor digitorum brevis (FDB) muscle fibers the biosensor was incorporated into the thin filaments, located between α-actinin labelled Z-lines (Fig. 1B,C). In transgenic mouse lines, the biosensor incorporated stoichiometrically into the sarcomere, replacing 64% of endogenous TnC in the myofilaments of intact, fast skeletal extensor digitorum longus (EDL) muscles (Transgenic (Tg): 64% ± 5% (n = 3), (Fig. 1D, Non-Transgenic (NTg) samples were included for visualization of endogenous TnC levels and were not used in the calculation of replacement values, see “Methods”).

**Figure 2:**
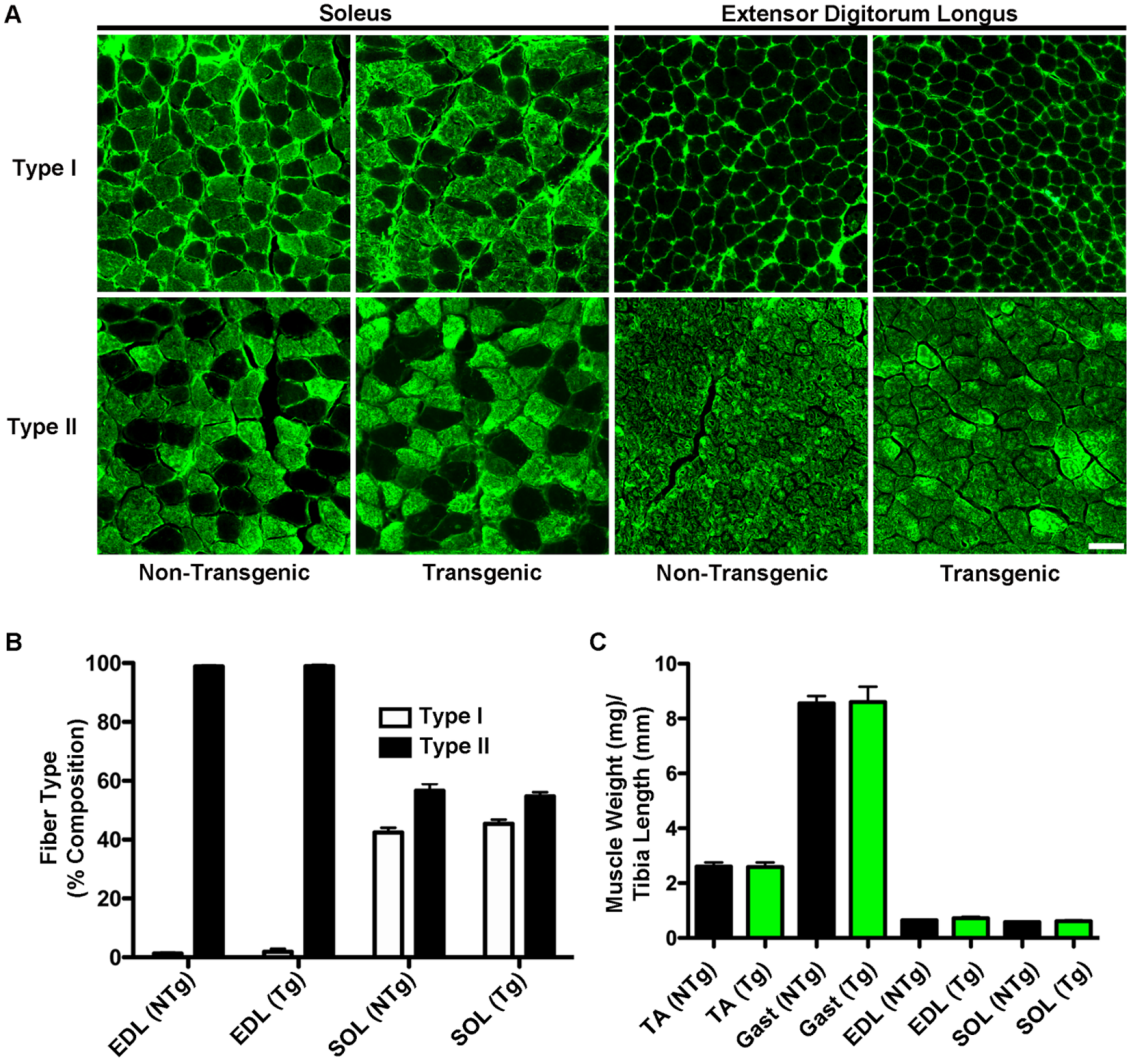
Expression of the fsTnC biosensor does not alter muscle weight or fiber type. (**A**) Immunofluorescence imaging of transverse muscle sections from extensor digitorum longus (EDL) and soleus (SOL) muscles from transgenic and non-transgenic animals stained with antibodies against Type I and Type II myosin heavy chain (scale bar = 50 μm). (**B**) Analysis of percentages of Type I and Type II fibers in EDL and SOL muscles from transgenic (n = 6) and non-transgenic (n = 6) animals. There is no significant difference between muscle type or genetic background. (**C**) Analysis of muscle weights as normalized to tibia length for tibialis anterior (TA), gastrocnemius (Gast), extensor digitorum longus (EDL), and soleus (SOL) muscles from transgenic (n = 10) and non-transgenic (n = 8) animals. There are no significant differences between muscle type or genetic background. Values are Mean ± S.E.M.

Incorporation of the fast skeletal biosensor did not alter skeletal muscle form or composition. Muscle weights were unchanged [TA: NTG: 2.602 ± 0.152 mg (n = 8), TG: 2.588 ± 0.164 mg (n = 10), GAST: NTG: 8.551 ± 0.274 mg (n = 8), TG: 8.606 ± 0.560 mg (n = 10), EDL: NTG: 0.644 ± 0.028 mg (n = 8), TG: 0.717 ± 0.057 mg (n = 10), SOL: NTG: 0.583 ± 0.023 mg (n = 8), TG: 0.609 ± 0.040 mg (n = 10)] and fiber type percentages [NTG: Type I: 1.26 ± 0.33% (n = 6), Type II: 98.79 ± 0.33% (n = 6), TG: Type I: 1.89 ± 0.99% (n = 6), Type II: 98.85 ± 0.47% (n = 6)] not different, as compared to non-transgenic controls (Fig. 2). Together these data demonstrate that the biosensor incorporates into the sarcomere via stoichiometric replacement of endogenous TnC, without cytosolic accumulation, and without impact on sarcomeric or overall muscle structure or composition.

### Biophysical analysis of the TnC biosensor

The design of the fsTnC biosensor is built on the foundation of extensive in vitro experimentation, which has previously demonstrated that FRET-based probes on TnC can detect calcium dependent conformational changes to TnC in reconstituted permeabilized preparations in steady-state conditions^19^. To characterize the novel TnC biosensor developed here, biochemical analysis of calcium dissociation rates and biophysical steady-state analysis of calcium titrations in permeabilized EDL muscle preparations were undertaken.

**Figure 3:**
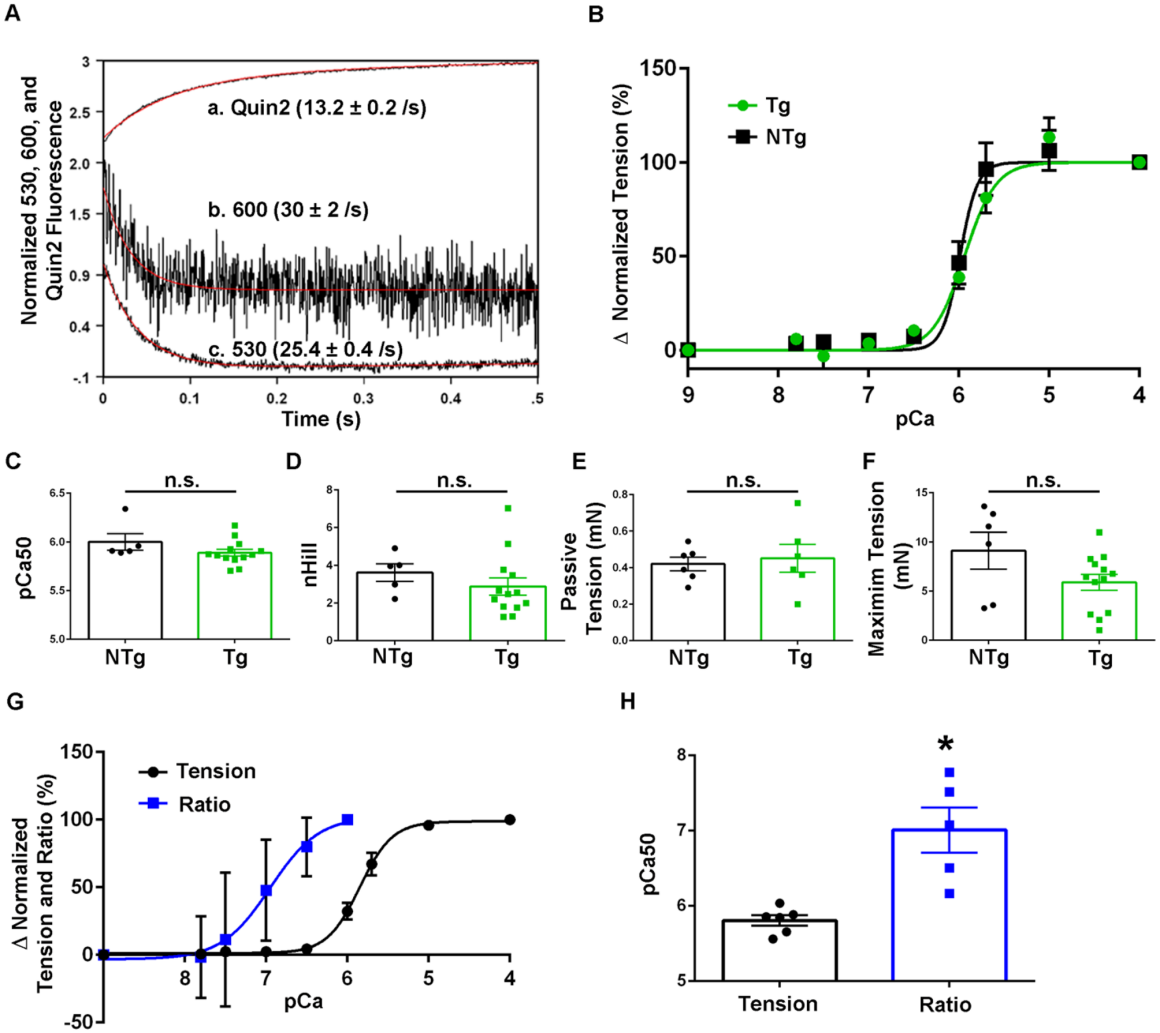
Biochemical and steady-state biophysical analysis of TnC biosensor. (**A**) Calcium dissociation rates of the N-terminal of the control TnC peptide using Quin2 (a) and the N-terminal of the TnC-FRET peptide using 600 nm wavelength for mRuby2 (b) and 530 nm wavelength for Clover (c). (**B**) Relative tension developed as skinned EDL muscle was exposed to solutions of increasing calcium concentrations. Non-transgenic ([NTg, (n = 5 muscles from n = 3 animals)] and transgenic [Tg, (n = 8 muscles from n = 4 animals)] were used. Tension was normalized to the pCa 4.0 value obtained in each preparation. (**C**) Summary statistics of calcium concentration at half-maximal activation (pCa50) for NTg and Tg muscles showed no significant differences. (**D**) Summary statistics of the Hill coefficient value (nHill) for NTg and Tg muscles showed no significant differences. (**E**) Summary statistics of passive tension measurements for NTg and Tg muscles showed no significant differences. (**F**) Summary statistics of max relative tension measurements for NTg and Tg muscles showed no significant differences. (**G**) Combined graph of relative tension and fluorescence ratio changes as skinned EDL muscle exposed to solutions of increasing calcium concentration. Tension was normalized to the pCa 4.0 value obtained in each preparation (n = 6 muscle halves from n = 2 animals) and ratio fluorescence was normalized to the pCa 6.0 value obtained in each preparation (n = 5 muscle halves from n = 2 animals). (**H**) Summary statistics of calcium concentration at half-maximal activation (pCa50) for relative tension and FRET ratio fluorescence showed a significant difference in the pCa50 values. (P < 0.05). Mean ± S.E.M. data are presented.

Calcium dissociation kinetics were examined using isolated recombinant TnC protein both with the FRET pair (TnC-FRET) and without as a control (TnC) (see “Methods”). The expression and purification protocol involved the addition of a 6-His tag on the C-terminal end of the protein products which remained throughout the following studies. At 20 °C, the rate of calcium dissociation from the N-terminal, regulatory domain, is > 1000/s and thus too fast to observe in this setting. In order to reveal the N-terminal regulatory sites I and II calcium off rates, complexity of the system was increased through the addition of the TnI (96–148) switch peptide, which is known to increase calcium sensitivity and slow calcium off rates of the N-terminal^24^. Here, when the calcium off rate of the N-terminal domain of both the control TnC and the TnC-FRET was examined under these conditions, it is seen that calcium dissociated from the N-terminal domain of the control TnC at approximately 13/s using Quin2, a fluorescent calcium chelator which reports the actual rate of calcium dissociation, (Quin2: 13.2 ± 0.2/s) (Fig. 3A). When the TnC-FRET donor was excited with 505 nM, and the fluorescence was monitored at 530 nm for the donor or 600 nm for the acceptor, both the donor and acceptor fluorescence decreased with a rate approximately 2 times faster than that of the control TnC (530 nM: 25.4 ± 0.4/s, 600 nM: 30 ± 2/s) (Fig. 3A). Thus, the function of the TnC-FRET biosensor is similar to that of the control TnC in the presence of the TnI (96–148) peptide. As has been previously shown, the interaction between TnC and TnI significantly increases the calcium sensitivity of TnC and this TnI (96–148) peptide in particular has been demonstrated to be important in regulating TnI interaction with actin and TnC, suggesting that the use of this peptide in this assay recapitulates native functions of the system and provides the necessary framework to study calcium interactions with TnC^24^.

In this analysis, it is important to note that due to the fluorescence of the FRET pair, alternative approaches were required to measure the calcium dissociation rates between the TnC control protein and the TnC-FRET protein, owing to spectral overlap via the FRET probes with the Quin-2 and Tyrosine measurements. Tyrosine is intrinsic to the TnC protein and thus is reporting environmental changes that the protein is undergoing^32^, while Quin-2 binds calcium as a direct reporter of calcium dissociation^33^. The fluorescence being measured with the TnC-FRET is driven by conformational changes occurring due to the dissociation of calcium from the protein complex. Within this simplified system, the addition of the stabilizing TnI(96–148) peptide not only allows for the resolution of the N-terminal kinetics, but it also demonstrates that the kinetics of the TnC-FRET system are close to the control condition (Fig. 3A) making them comparable, and suggesting the TnC biosensor is an excellent probe to follow calcium exchange kinetics in the muscle. From this data, as well as previous studies^24,25,34,35^, it is expected that the fidelity of the biosensor, and its ability to detect FRET, will be further enhanced as the complexity of the muscle system is increased, as shown in past reports^36^.

### Permeabilized skeletal muscle

To continue to advance the physiological complexity of the experimental system to evaluate any impact of the biosensor fluorescence probe on the function of the TnC protein, isolated, permeabilized EDL muscles from transgenic animals, expressing the biosensor, and non-transgenic littermate control animals, not expressing the biosensor, were used in steady-state activating conditions. Tension-pCa relationships were obtained by recording force changes during steady-state calcium titrations (Fig. 3B). The results demonstrated no statistically significant differences in pCa50 [NTg: 6.00 ± 0.09 (n = 5), Tg: 5.89 ± 0.03 (n = 13)] (Fig. 3C) or the nHill coefficient [NTg: 3.62 ± 0.46 (n = 5), Tg: 2.88 ± 0.46 (n = 13)] (Fig. 3D). Passive tension was measured as the difference between baseline tension at optimum length (L_o_) and at L_o_—10% and showed no significant difference between non-transgenic and transgenic muscle [NTg: 0.42 ± 0.04 mN (n = 6), Tg: 0.45 ± 0.08 mN (n = 6)] (Fig. 3E). Additionally, there was no significant difference between maximum relative tension of non-transgenic and transgenic muscles [NTg: 9.12 ± 1.88 mN (n = 6), Tg: 5.9 ± 0.81 mN (n = 13)] (Fig. 3F). Taken together this provides evidence that the incorporation of the biosensor into the sarcomeres had no significant effect on the steady state calcium activating function of the muscle.

Next, steady-state force and fsTnC FRET signals in permeabilized EDL muscle were simultaneously measured while calcium titrations were conducted. Reproducible steady-state FRET ratio values were obtained for calcium titrations between pCa9 and pCa6. At high steady-state calcium titrations, significant motion of the EDL preps, which moved the muscle in and out of optical focus, precluded reliable steady-state FRET ratio measurements. Nonetheless, the results demonstrate sigmoidal tension-pCa and fsTnC FRET-pCa relationships, in agreement with previous works^19,22^. This suggests that the biosensor was able to detect calcium-driven conformational changes in TnC in a calcium concentration dependent manner (Fig. 3G). This is seen through the steady-state increase in FRET in direct relation to the increased activating levels of calcium. Under steady-state calcium activating conditions, summary data show the TnC FRET ratio-pCa curve is markedly left-shifted as compared to the tension-pCa curve, resulting in significantly different pCa50 values between force and FRET ratio (pCa50: Tension: 5.81 ± 0.07 (n = 6), Ratio: 7.01 ± 0.30 mN (n = 5) (Fig. 3H). This demonstrates that the steady-state biosensor FRET signal was significantly more sensitive to calcium than isometric tension.

### TnC biosensor transient in intact FDB single fibers

**Figure 4:**
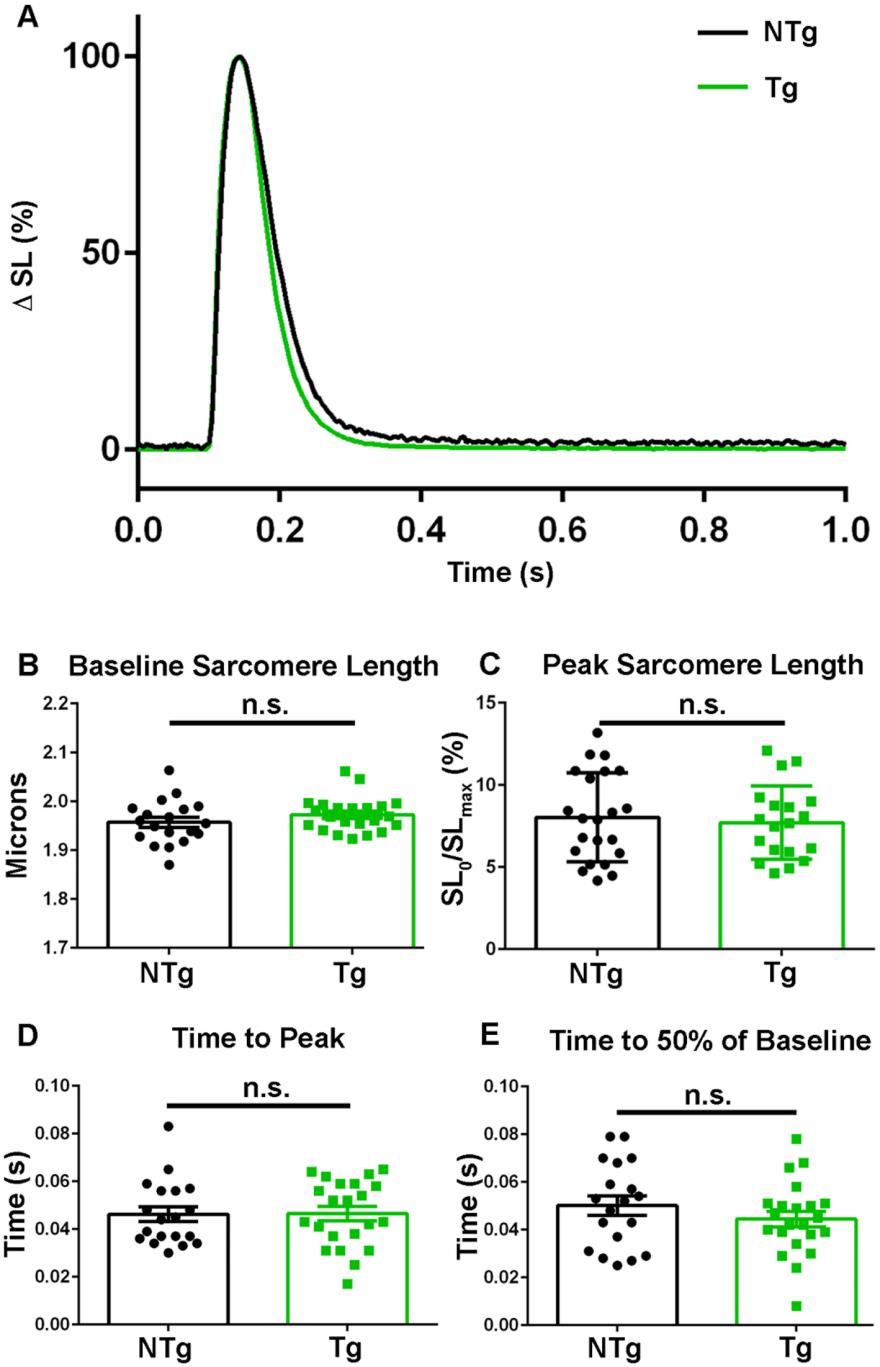
TnC biosensor expression and integration does not impact sarcomere function during a twitch contraction. (**A**) Ensemble average of normalized (0–100%) amplitude of sarcomere length changes during a single twitch contraction for non-transgenic (black) and transgenic (green) isolated FDB fibers. (**B**) Summary statistics of baseline sarcomere length from non-transgenic and transgenic isolated FDB fibers show there is no significant difference between groups. (**C**) Summary statistics for the peak sarcomere length from non-transgenic and transgenic isolated FDB fibers demonstrate there is no significant difference between groups. (**D**) Summary statistics for the time to peak from non-transgenic and transgenic isolated FDB fibers demonstrate there is no significant difference between groups. (**E**) Summary statistics for the time to 50% of baseline in isolated FDB fibers from non-transgenic and transgenic animals show no significant difference between groups. Myofibers measured at room temperature with 0.2 Hz stimulation, NTg: n = 19 fibers from n = 3 animals and Tg: n = 26 fibers from n = 7 animals. Mean ± S.E.M. are presented, *P < 0.05.

To further advance physiological relevance of the TnC biosensor, real time FRET recordings were obtained in live FDB fibers during a twitch contraction. Dynamic sarcomere length changes were obtained in isolated FDB myofibers from transgenic mice expressing the biosensor and in non-transgenic littermate controls (Fig. 4A,B). Data showed no significant differences in the time to peak of sarcomere length change [NTg: 0.05 ± 0.003 s (n = 19), Tg: 0.05 ± 0.003 s (n = 22)] (Fig. 4C) or in the relaxation kinetics [Time to 50% of Baseline: NTg: 0.05 ± 0.004 s (n = 19), Tg: 0.04 ± 0.003 s (n = 22)] between transgenics and controls (Fig. 4D). Additionally, contractility of the isolated FDBs was unaffected with no significant differences between non-transgenic and transgenic myofibers in regards to baseline sarcomere length [NTg: 1.96 ± 0.01 s (n = 19), Tg: 1.97 ± 0.008 s (n = 22)] (Fig. 4C) or percent change from baseline [NTg: 7.71 ± 0.51 s (n = 19), Tg: 8.04 ± 0.58 s (n = 22)] (Fig. 4). This, along with previously discussed data relating to the muscle weight and muscle fiber composition (Fig. 2) and steady-state tension-pCa data (Fig. 3), provides direct physiological evidence that the expression and incorporation of the TnC biosensor into sarcomeres allows for normal TnC function in intact muscle fibers during twitch contractions.

**Figure 5:**
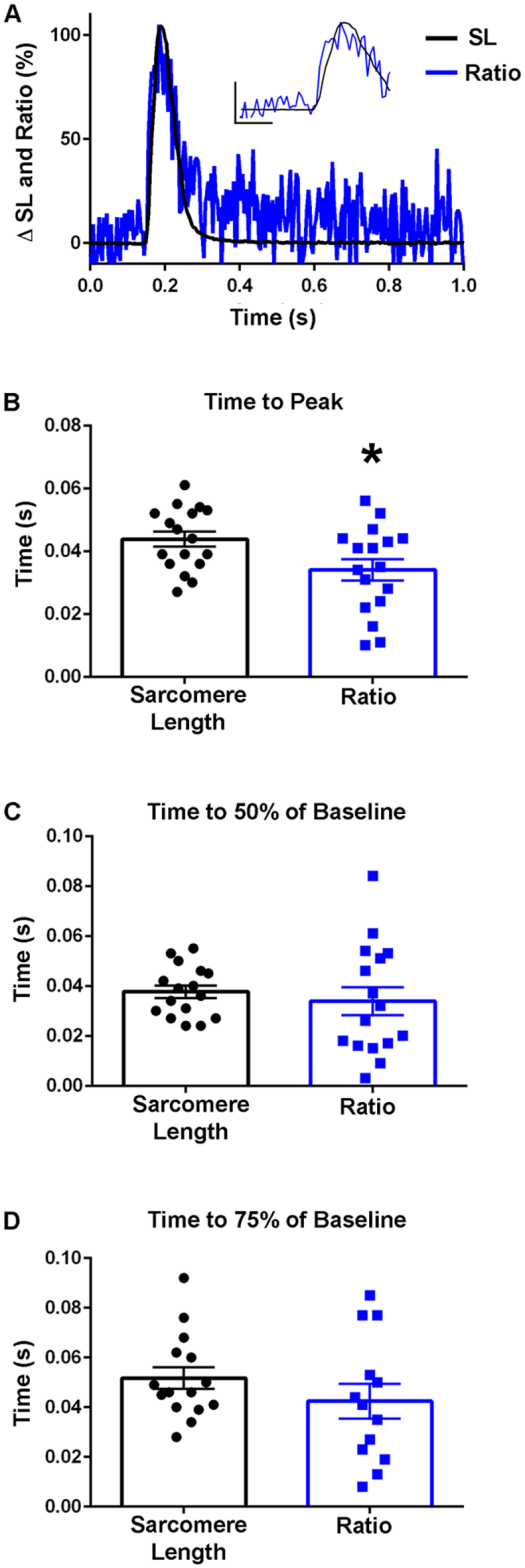
TnC biosensor transient in unloaded intact FDB myofibers at room temperature. (**A**) Normalized traces (0–100%) of an ensemble average of biosensor ratio fluorescence (blue) and sarcomere length (black) dynamics which demonstrates the biosensor ratio transient peak precedes the peak of the sarcomere length transient. Inset shows same data and serves to highlight the differences of the peak timing (y-axis represents a 50% change from baseline, x-axis represents 0.10 s). (**B**) Summary statistics for time to peak for the biosensor ratio and sarcomere length indicate peak activation of the biosensor ratio (0.0341 ± 0.0034 s) occurred significantly before the peak sarcomere length change (0.0438 ± 0.0024) s (n = 17 myofibers from 11 animals in each group). (**C**) Summary statistics for the time to 50% of baseline for the biosensor ratio and sarcomere length demonstrated no significant difference between the ratio or sarcomere length (n = 16 myofibers from n = 11 animals in each group). (**D**) Summary statistics for the time to 75% of baseline for the biosensor fluorescence ratio and sarcomere length changes showed no significant difference in the relaxation dynamics of the biosensor and sarcomere length (Sarcomere length: n = 15, Ratio: n = 13 from n = 9 animals). Myofibers measured at room temperature with 0.2 Hz stimulation. Mean ± S.E.M. are presented, *P < 0.05.

To further build upon the biochemical and steady state FRET biosensor studies (Fig. 3), FDB myofibers from transgenic mice were isolated and upon stimulation changes in biosensor fluorescence and sarcomere length were determined simultaneously (Fig. 5A). Due to the length of FDB myofibers (~ 500–700 μm)^37^ it is not possible to collect all the emitted fluorescence across the entire contracting myofiber with the imaging system. Thus, to account for motion effects during twitch contractions mRuby2/Clover ratios were necessary to track the biosensor transient (see “Methods”). This is a well-validated and accepted method to address motion artifacts in larger muscles^30^. As the design of the biosensor employed the use of an intramolecular FRET pair, motion will impact an equal amount of Clover and mRuby2 fluorophores. This means the ratio will be able to account for any fluorescence due to motion, leaving only fluorescence changes that are exclusively due to contraction-mediated conformational changes in the TnC-FRET biosensor. Additionally, previous work has shown that when CFP/YFP donor and acceptor fluors are separated by a long linker region, resulting in donor and acceptor fluor being unable to interact, there is no consistent change in FRET when calcium is added. This provides evidence that the binding of a divalent cation, such as calcium, is not enough to change the emission of the fluors^19^.

**Figure 6:**
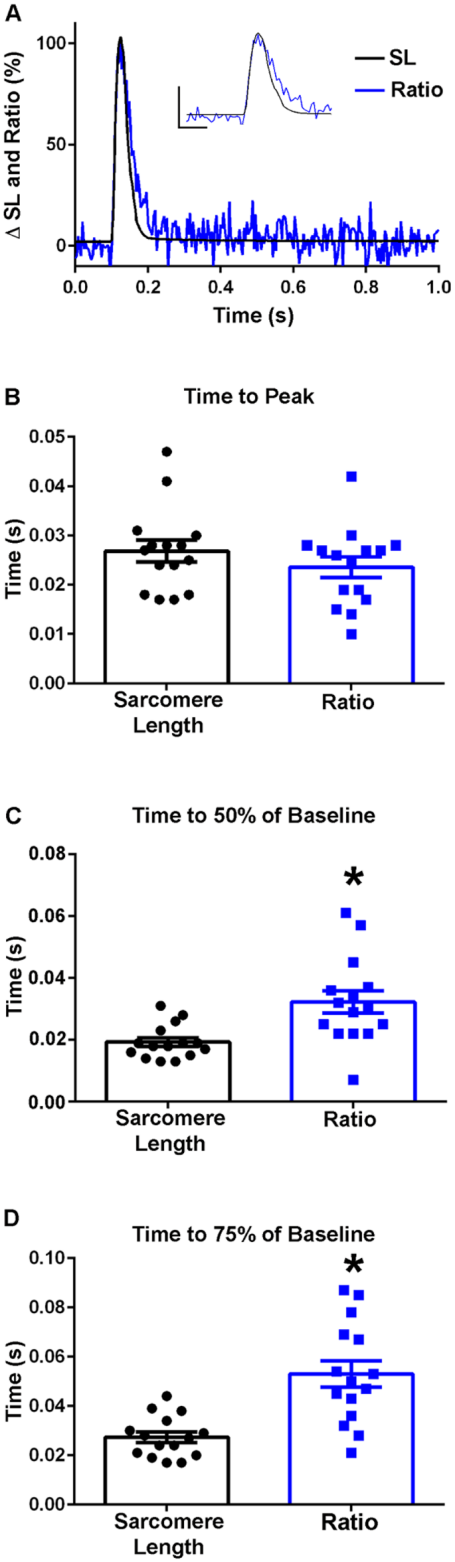
TnC biosensor transient in intact FDB myofibers at 37 °C. (**A**) Normalized traces (0–100%) of an ensemble average of biosensor ratio fluorescence (blue) and sarcomere length (black) dynamics. Inset shows same data and serves to highlight the region of the peak amplitude (y-axis represents a 50% change from baseline, x-axis represents 0.05 s). (**B**) Summary statistics for time to peak for the biosensor ratio and sarcomere length shows timing of the peak activation of the biosensor ratio is not significantly different from the time to peak of the sarcomere length (n = 15 myofibers in each group from n = 7 animals). (**C**) Summary statistics for the time to 50% of baseline for the biosensor ratio and sarcomere length demonstrate that biosensor inactivation (0.0323 ± 0.0036 s) was delayed relative to the relaxation of the myofiber (0.0193 ± 0.0014 s) (n = 15 myofibers in each group from n = 7 animals). (**D**) Summary statistics for the time to 75% of baseline for the biosensor fluorescence ratio and sarcomere length showed a significant difference in the relaxation dynamics of the biosensor and sarcomere length, with the biosensor inactivation (0.0530 ± 0.0053 s) slower than the sarcomere length relaxation of the myofiber (0.0274 ± 0.0022 s) (n = 15 myofibers in each group from n = 7 animals). Myofibers measured at 37 °C with 0.2 Hz stimulation. Mean ± S.E.M. are presented, *P < 0.05.

At room temperature, in intact, single FDBs, the biosensor Clover and mRuby2 signals were anti-correlated, indicating FRET. Importantly, these fluorescence signals were resolved in the milliseconds timescale, which is the physiological time domain of a single skeletal muscle twitch. This result, along with findings in the permeabilized system (Fig. 3), demonstrates that the biosensor can report the global conformational changes of TnC that occur in the time domain of a single physiological twitch. At room temperature, the results indicate that the biosensor ratio transient reached its peak amplitude significantly faster than the peak amplitude of sarcomere length changes (Fig. 5A,B). These findings in intact muscle fiber data can be understood in the context of conformational changes in TnC which occur upon calcium binding, which precedes muscle contraction^1,38^. Experiments were also performed at 37 °C, demonstrating that the kinetics of the biosensor are sensitive to temperature, at a level similar to those changes seen in sarcomere length changes (Fig. 6A,B). These changes are in line with effects of temperature on muscle function as previously described which includes temperature effects on the troponin regulatory system in skeletal muscle, intracellular calcium release and reuptake, and actomyosin ATPase activity and cross-bridge formation, where the speed of contraction development is increased at higher temperatures^1,39,40^. Interestingly, at 37 °C, significant differences became apparent during relaxation, wherein the inactivation of the TnC biosensor transient was significantly slower than sarcomere relengthening (Fig. 6C,D), which was not seen in experiments done at room temperature (Fig. 5C,D).

**Figure 7:**
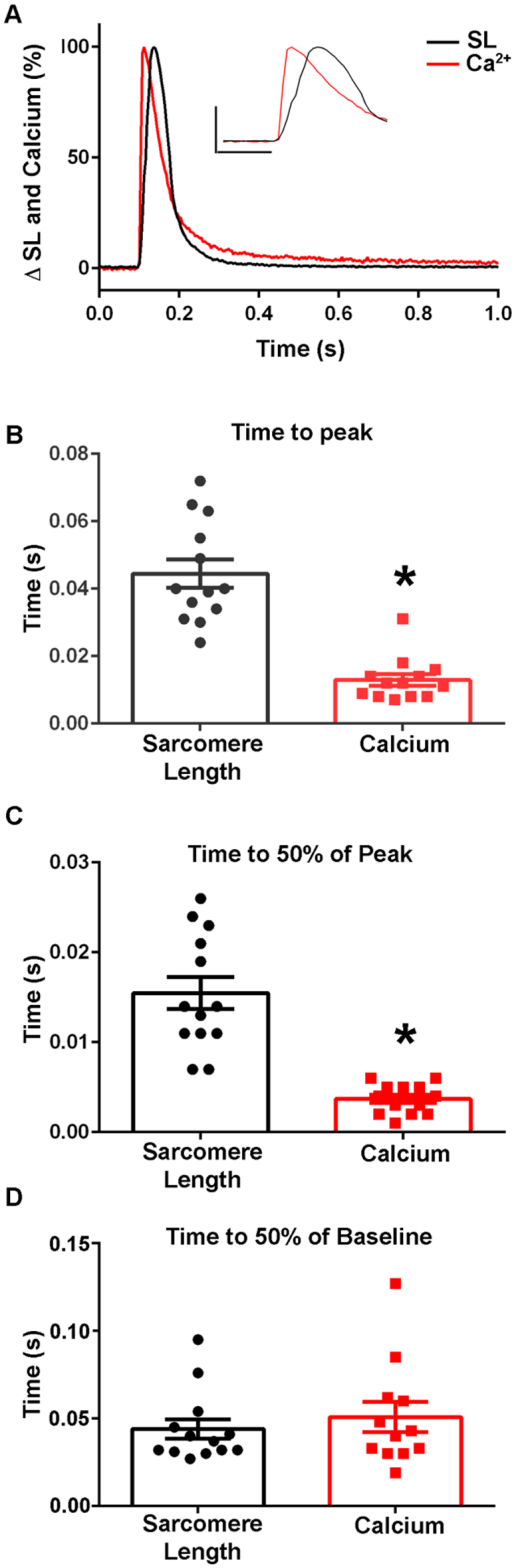
Calcium dynamics in isolated intact FDB myofibers at room temperature. (**A**) Normalized traces (0–100%) of an ensemble average of FURA-2 calcium fluorescence (red) and sarcomere length (black) dynamics showing that the calcium transient time to peak precedes the peak of the sarcomere length transient. Inset shows same data and serves to highlight the differences of the peak timing (y-axis represents a 50% change from baseline, x-axis represents 0.10 s). (**B**) Summary statistics for time to peak for the calcium transient and sarcomere length changes shows peak amplitude of the calcium transient occurs significantly before the peak sarcomere length change (n = 13 myofibers in each group from n = 3 animals). (**C**) Summary statistics for the time to 50% of peak for the calcium transient and sarcomere length demonstrates there is a significant difference between the calcium kinetics (0.0037 ± 0.0005 s) and sarcomere length changes (0.0155 ± 0.0018 s) (n = 13 myofibers in each group from n = 3 animals). (**D**) Summary statistics for the time to 50% of baseline for the calcium transient (0.0508 ± 0.0087 s) and sarcomere length changes (0.0440 ± 0.0056 s) shows there is no significant difference in the recovery dynamics of calcium and sarcomere length relaxation (Sarcomere length: n = 13, FURA-2: n = 12 from n = 3 animals). Myofibers measured at room temperature with 0.2 Hz stimulation. Mean ± S.E.M. are presented, *P < 0.05.

**Figure 8:**
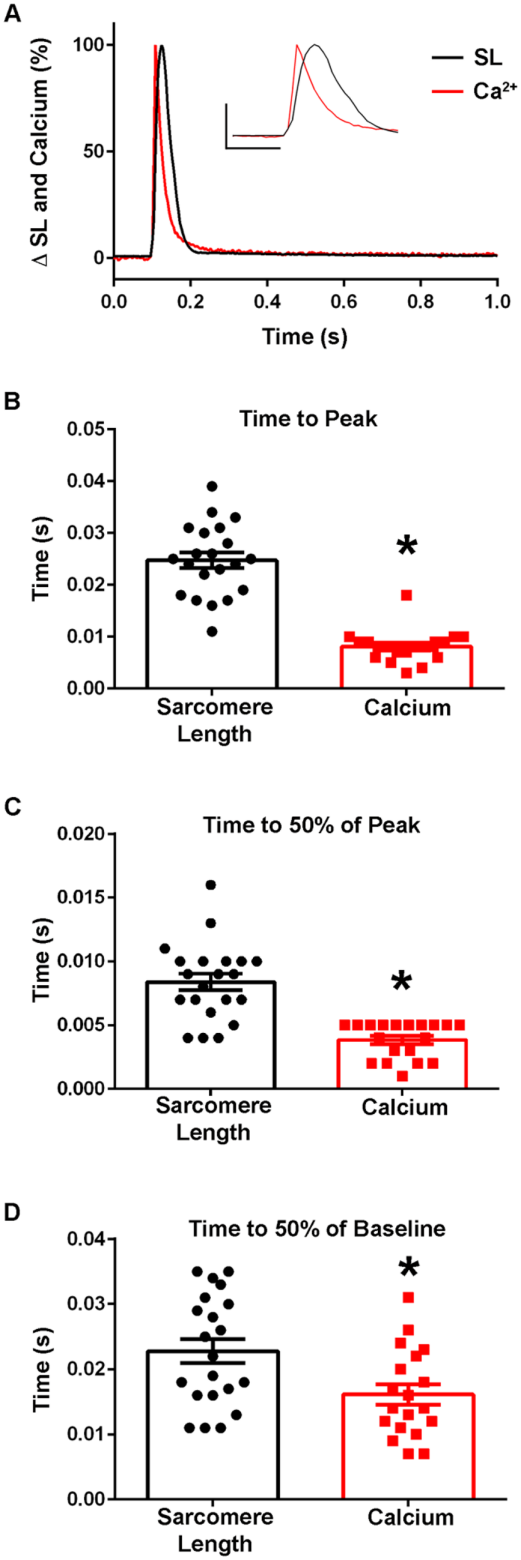
Calcium dynamics in isolated intact FDB myofibers at 37 °C. (**A**) Normalized traces (0–100%) of an ensemble average of FURA-2 calcium fluorescence (red) and sarcomere length (black) dynamics. Inset shows same data and serves to highlight the region of the peak amplitude (y-axis represents a 50% change from baseline, x-axis represents 0.10 s). (**B**) Summary statistics for time to peak of the calcium transient and sarcomere length changes shows timing of the peak calcium amplitude occurs significantly before the time to peak of the sarcomere length (Sarcomere length: n = 21, FURA n = 19 from n = 2 animals). (**C**) Summary statistics for the time to 50% of peak for the calcium transient and sarcomere length demonstrates that the calcium transient (0.0038 ± 0.0003 s) is significantly faster than the sarcomere length change (0.0084 ± 0.0006 s) (Sarcomere length: n = 21, FURA n = 19 from n = 2 animals). (**D**) Summary statistics for the time to 50% of baseline for the FURA-2 calcium transient and sarcomere length changes shows there is a significant difference in the recovery dynamics of the calcium transient and sarcomere length, with the calcium transient (0.0161 ± 0.0015 s) recovering more quickly than the relaxation of the myofiber (0.0228 ± 0.0018 s) (Sarcomere length: n = 21, FURA n = 19 from n = 2 animals). Myofibers measured at 37 °C with 0.2 Hz stimulation. Mean ± S.E.M. are presented, *P < 0.05.

Previous work has resulted in the design of cellular biosensors able to monitor intracellular calcium dynamics, but not twitch driven changes in dynamic sarcomere activation^41,42^. To determine that the novel biosensor designed here is not simply tracking changes in intracellular calcium concentration, intracellular calcium transients were examined (Figs. 7, 8) These experiments were performed in single FDB fibers isolated from non-transgenic animals to prevent spectral overlap between the calcium indicator Fura-2 and the excitation and emission spectra of the fluorophores of the biosensor. At both room temperature and at 37 °C in these myofibers, the cytoplasmic calcium transient time to peak was significantly faster than that of the sarcomere length [RT: SL: 0.05 ± 0.004 s (n = 13), FURA: 0.01 ± 0.002 s (n = 13), 37 °C: SL: 0.03 ± 0.002 s (n = 21), FURA: 0.008 ± 0.001 s (n = 19)] (Figs. 7A,B, 8A,B), results anticipated based on the known intracellular dynamics of muscle contraction^1,38^.

**Figure 9:**
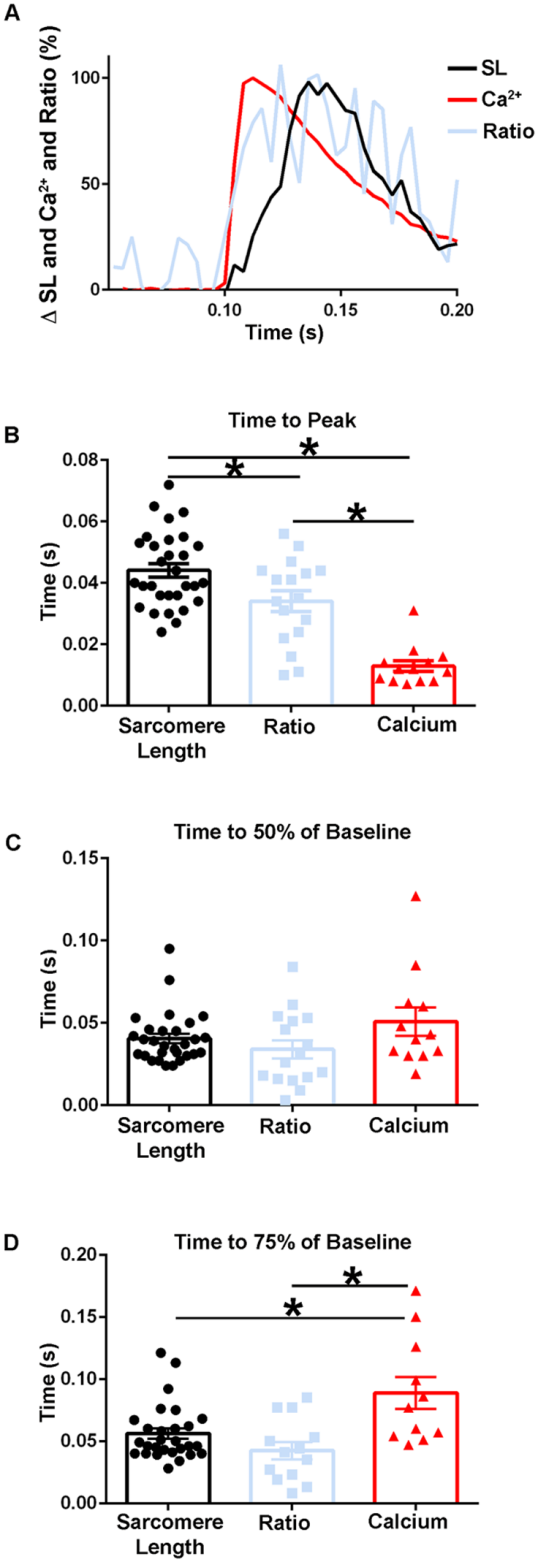
Temporal alignment of sarcomere length, TnC biosensor transient, and calcium transient kinetics during a twitch at room temperature. (**A**) Normalized traces (0–100%) of an ensemble average of FURA-2 calcium fluorescence (red), biosensor ratio fluorescence (blue), and sarcomere length (black) dynamics. This figure is a graphic compilation of data shown in Figs. 6 and 8. (**B**) Summary statistics of the time to peak amplitude for sarcomere length, biosensor transient, and calcium transient shows the time to peak of the biosensor significantly precedes that of the sarcomere length, and the time to peak of calcium is reached before both the biosensor transient and sarcomere length (Sarcomere length: n = 30 myofibers from n = 14 animals, Ratio: n = 17 myofibers from n = 11 animals, FURA-2: n = 13 myofibers from n = 3 animals). (**C**) Summary statistics of the time to 50% of baseline for sarcomere length, biosensor ratio transient, and the calcium transient (Sarcomere length: n = 29 myofibers from n = 14 animals, Ratio: n = 16 myofibers from n = 11 animals, FURA-2: n = 12 myofibers from n = 3 animals). (**D**) Summary statistics for the time to 75% of baseline for sarcomere length, biosensor ratio transient, and calcium transient shows the calcium decay transient is significantly slower than both the relaxation of sarcomere length and the biosensor transient inactivation. (Sarcomere length: n = 29 myofibers from n = 14 animals, Ratio: n = 16 myofibers from n = 11 animals, FURA-2: n = 12 myofibers from n = 3 animals). Myofibers measured at room temperature with 0.2 Hz stimulation. Mean ± S.E.M. are presented, *P < 0.05.

**Figure 10:**
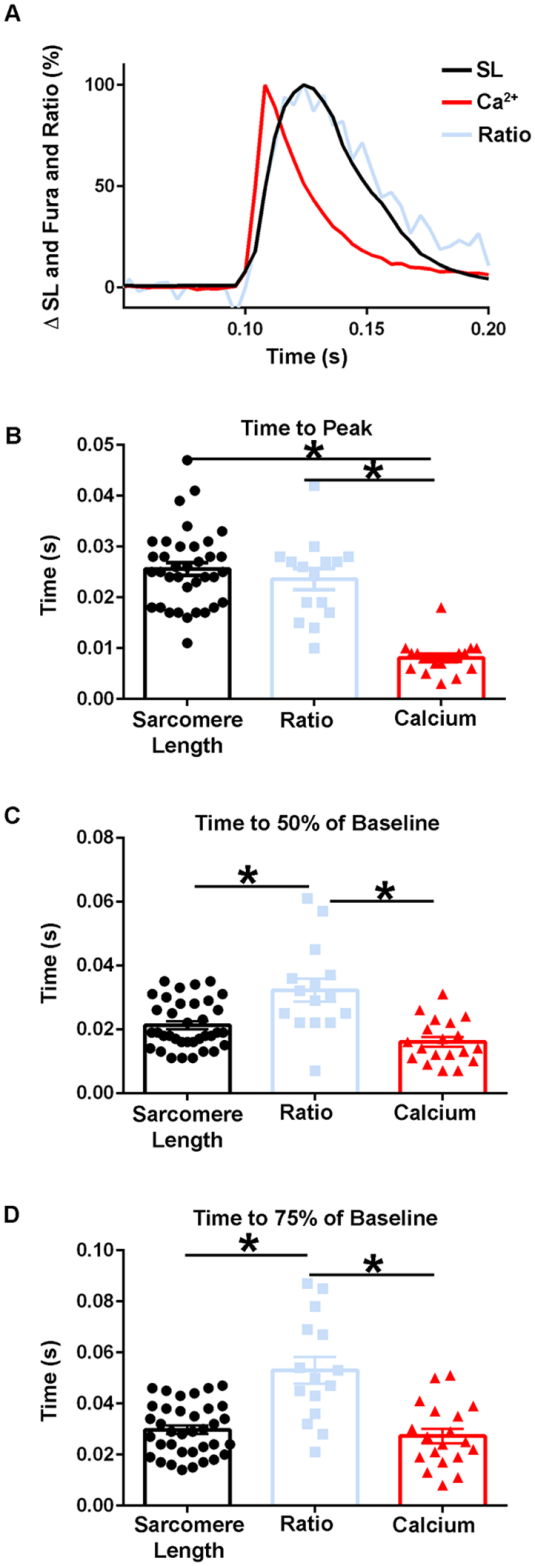
Temporal alignment of sarcomere length, biosensor transient, and calcium transient kinetics in intact FDB fibers during a twitch at 37 °C. (**A**) Normalized traces (0–100%) of an ensemble average of FURA-2 calcium fluorescence (red), biosensor ratio fluorescence (blue), and sarcomere length (black) dynamics. This figure is a compilation of data shown in Figs. 7, 9. (**B**) Summary statistics of the time to peak amplitude for sarcomere length, biosensor transient, and calcium transient (Sarcomere length: n = 36 myofibers from n = 9 animals, Ratio: n = 15 myofibers from n = 7 animals, FURA-2: n = 19 myofibers from n = 2 animals). (**C**) Summary statistics of the time to 50% of baseline for sarcomere length, biosensor ratio transient, and the calcium transient show the biosensor transient inactivation is significantly slower than the relaxation of sarcomere length and the recovery of the calcium transient (Sarcomere length: n = 36 myofibers from n = 9 animals, Ratio: n = 15 myofibers from n = 7 animals, FURA-2: n = 19 myofibers from n = 2 animals). (**D**) Summary statistics for the time to 75% of baseline for sarcomere length, biosensor ratio fluorescence transient, and calcium transient shows the calcium decay transient is significantly faster than the biosensor transient inactivation, while biosensor inactivation is significantly slower than the relaxation of the sarcomere length. (Sarcomere length: n = 36 myofibers from n = 9 animals, Ratio: n = 15 myofibers from n = 7 animals, FURA: n = 19 myofibers from n = 2 animals). Myofibers measured at 37 °C with 0.2 Hz stimulation. Mean ± S.E.M. are presented, *P < 0.05.

Importantly, when the time to peak of the calcium transient was compared to the time to peak of the TnC biosensor ratio, the dynamics of the fluorescence signal differed significantly, with the time to peak of the calcium transient preceding that of the ratio (Figs. 9A,B, 10A,B). There were also marked differences seen between the decay kinetics of the calcium and ratio transients. Notably the times to 50% of baseline and the time to 75% of baseline for the ratio were significantly slower than that of the calcium transient in experiments done at 37 °C [Time to 50% of Baseline: Ratio: 0.03 ± 0.004 s (n = 15), FURA: 0.02 ± 0.002 s (n = 19), Time to 75% of Baseline: Ratio: 0.05 ± 0.005 s (n = 15), FURA: 0.03 ± 0.003 s (n = 19)] (Fig. 10C,D) and not in those done at room temperature (Fig. 9C). Additionally, as Fura-2 is a high affinity calcium dye^43^, the decay kinetics of the transient are overestimated and even faster than reported. Together, this indicates that the biosensor is not simply a calcium detector and is instead able to report the activation of the sarcomere as mediated through calcium-TnC interactions.

## Discussion

We report here the design, development, and implementation of a new skeletal muscle sarcomere activation biosensor permitting, for the first time to our knowledge, real time investigations of dynamic sarcomere performance in live single skeletal muscle fibers. The biosensor design features an intramolecular, high fidelity FRET pair, composed of Clover and mRuby2, integrated into the N- and C-termini of human fast skeletal troponin C, respectively. Main new findings include the biochemical and biophysical characterization of the purified TnC biosensor demonstrating evidence of FRET upon Ca^2+^ binding and showing Ca^2+^ binding kinetics comparable to purified WT human fsTnC, evident upon reconstitution with the fast skeletal TnI (96–148 aa) switch peptide. In transgenic mice with skeletal muscle-directed expression of the fsTnC biosensor, data show myofilament localization with stoichiometric incorporation of the TnC biosensor in the sarcomere. In the TnC biosensor-expressing animals, skeletal muscle morphology, fiber type, and contractile function were not altered by incorporation of the TnC biosensor transgene. In permeabilized EDL muscles studied under steady-state conditions, there were no significant differences in the pCa50 values between the biosensor expressing transgenic animals and their non-transgenic littermate controls. Interestingly, in transgenic muscles, the Ca^2+^-activated TnC biosensor signal was significantly left-shifted as compared to the Ca^2+^-activated tension. Importantly, in intact single adult skeletal muscle fibers, real time twitch contractile data showed the TnC biosensor transient preceding the peak of contraction. Further, under the physiological temperature of 37 °C, inactivation of the TnC biosensor transient decayed significantly more slowly than the Ca^2+^ transient and contraction. The uncoupling of the biosensor transient from the Ca^2+^ transient indicates the biosensor is not functioning as a Ca^2+^ transient reporter per se, but rather reports myofilament activation via conformation of TnC that, in turn, has been determined to reflect the ensemble effects of multiple activating ligands within the sarcomere^1^. With this new system, data show the physiologically relevant temporal relationship between the intracellular Ca^2+^ transient, sarcomere activation, via the TnC biosensor, and contractile function all in real time in live fast twitch single skeletal muscle fibers. We propose this new system will be useful in revealing the complexities of physiological sarcomere activation in healthy and diseased skeletal muscle fibers.

### Interpretation of the TnC biosensor transient in live skeletal muscle fibers

Data show the TnC biosensor transient is capable of reporting the dynamic state of sarcomere activation within the context of an intact skeletal muscle fiber during the time course of a live skeletal muscle fiber twitch contraction. This interpretation is built on the premise that TnC serves as a nexus for reporting effects of multiple inter-myofilament signaling ligands in determining the overall activation status of the sarcomere^1,6,8,10–12^, as discussed in more detail below. Previous work, featuring an isolated TnC-FRET molecule in an in vitro system, directly demonstrated a Ca^2+^-dependent TnC structural compaction^19^. This result is in keeping with a distinct TnC structural change requisite for overall thin filament regulation^6,8^. The present work shows the TnC FRET-based biosensor incorporates normally into the sarcomere where it functions similarly to wild type TnC in the context of intact skeletal muscle fibers. Conceptually, this is substantiated by previous works using similar genetic engineering approaches to strategically modify key thin and thick myofilament proteins of the sarcomere^44^. We therefore interpret the TnC biosensor FRET transient as reporting TnC’s global conformational change during contraction, from an initial extended state (relaxed) to a compacted state (activated) and back to an extended state (relaxed), during the timescale of a physiological twitch, as based on extensive in vitro work on TnC, including TnC FRET^19^.

More specifically, in analyzing the TnC biosensor twitch transient upon electrical stimulation of the muscle fiber, we propose that the sarcomere integrated TnC-biosensor Clover and mRuby2 fluorophores come closer together, leading to the rise of the FRET transient observed. In the context of global conformational changes reported in TnC during activation by Ca^2+^^19^, the R_0_ for Clover–mRuby2 FRET pair, defined as the distance at which 50% efficiency of energy is transferred from Clover to mRuby2, is 6.3 nm^17,18^. Thus, we can infer from the TnC biosensor FRET transient that the spatial positioning of the Clover and mRuby2 intramolecular probes must be near the R_0_ (6 nm) to be able to detect the biosensor FRET signal that is observed during contraction. As the myofilament integrated TnC biosensor transitions to a compacted conformation and then back to extended state during the twitch, this can account for the millisecond time-scale rise and then fall to baseline observed in the TnC biosensor FRET transient we have obtained. During a twitch, as based on the Clover/mRuby2 R_0_ of 6.3 nm, together with knowledge of the useful FRET pair spacing range of 0.5–1.5 R_0_^17,18,45^, we can estimate that the intramolecular Clover to mRuby2 fluorophore distance varies during the twitch from about 3 nm, at the peak of the FRET transient, to a distance extending out to >  ~ 9 nm, accounting for the FRET transient decay and signal return to baseline. This interpretation does not require measuring the precise distances between the fluorophores during a contraction. Rather, our interpretation of the FRET transient relies on the above FRET pair distance parameters as sufficient in concluding the Clover and mRuby2 spatial juxtaposition must vary within this near R_0_ range in order to record the FRET transient observed.

It is also important to note that the TnC biosensor is stoichiometrically integrated into the sarcomere as consistent with the spacing of the troponin regulatory complex located at every seven actin monomers along the thin myofilament^8^. Thus, based on the R_0_ value of Clover/mRuby2, we can further surmise that each TnC biosensor operates independently from neighboring biosensors. In this interpretation, the biosensor transient reflects only intramolecular FRET and not inter-molecular FRET across the 3D space of the sarcomeres aligned in the fiber. It also follows that this sarcomere-integrated TnC biosensor differs substantially from other FRET probes specifically designed as cytoplasmic or myofilament localized Ca^2+^ indicators^46,47^.

To address concerns of potential motion artifacts that might obscure interpretation of the TnC biosensor transient, we used a validated ratiometric approach, using the ratio of the time-matched mRuby2/Clover transients^30^, for reporting the TnC FRET biosensor transient. Moreover, we report data for the TnC biosensor in terms of FRET intensities, which is an acceptable standard in the field, as discussed previously^22^. Considering that the number of Clover/ mRuby2 fluorophores varies in the optical detection spatial volume as a function of time, and from individual fiber to fiber, it would be technically challenging to accurately measure FRET efficiency in this system or determine with precision the Clover to mRuby2 probe distances. We posit that even if accurate FRET efficiency measurements were possible, they would not add significant new information regarding the interpretation of these FRET intensity-based findings. This is because our interpretation depends primarily on the direct association between the time-dependence of TnC biosensor conformational changes in concert with the time dependence of the Ca^2+^ transient and muscle fiber contraction records in response to a single electrical stimulation. From this discussion, we conclude that the changes in FRET reported here are within the expected range for the Clover- mRuby2 pair integrated into TnC^48,49^. We can further deduce that the fluorescence signals obtained here are of excellent spatial precision sufficient to detect the dynamic sub-nanometer changes in the FRET pair intramolecular distance that are occurring during contraction of an intact skeletal muscle fiber. Accordingly, it then follows that the time dependence of the TnC FRET transient provides a highly accurate record of the actual time dependence of TnC structural change that occurs during a fast skeletal muscle contraction. Taken together, we are reporting accurately the time sensitive conformational changes in TnC that arise during the msec time domain of a physiological twitch.

### Temporal alignment of the TnC biosensor transient with the Ca^2+^ transient and contraction

Analysis of TnC FRET biosensor transient kinetics in temporal alignment with the intracellular Ca^2+^ transient and contractile dynamics reveals in live skeletal muscle fibers the time sensitive, physiological mechanism of mammalian skeletal muscle fiber sarcomere activation. Sequentially, at physiological temperatures, data show that the Ca^2+^ transient rises and significantly decays prior to the attainment of the peak of the TnC biosensor transient. In addition, at 37 °C, the inactivation of the TnC biosensor transient is significantly prolonged relative to the Ca^2+^ transient and for contraction. This is evident in plotting the times to 50% and 75% return to baseline wherein the TnC biosensor times are significantly slower than that of the Ca^2+^ transient recovery back to baseline. Interestingly, data also show that the TnC biosensor transient decays more slowly than the time course of muscle fiber relaxation. At room temperature conditions, it is further evident that the TnC biosensor transient attains peak value prior to the contractile peak. Taking these together, these data indicate the TnC biosensor is not a reporter of the intracellular Ca^2+^ transient, but rather reflects the ensemble of myofilament regulatory inputs which are known to influence TnC’s regulatory conformation. From detailed biophysical studies the TnC biosensor inputs include, the N-terminal TnC-Ca^2+^ complex, the troponin and tropomyosin regulatory complex, and myosin cross-bridge binding, cycling rates and activation status^1–3,6,8,50–52^. It will be interesting in future studies to leverage this new system to dissect the specific regulatory roles of each of these inputs during a live twitch contraction in skeletal muscle fibers.

Our findings find support from previous works modeling the rates of Ca^2+^-activated myofilament regulatory states in intact muscle systems. In silico studies have postulated that changes in the conformation of myofilament components, including troponin, tropomyosin alignment on the actin thin filament, and myosin cycling, rise and decay in sequence following the effects of Ca^2+^ initially binding to troponin during a theoretical twitch contraction^53^. Moreover, earlier studies have featured low angle X-ray diffraction technology to probe tropomyosin’s azimuthal regulatory position during twitch contractions in intact amphibian skeletal muscles^54^. Here, during the time course of a single twitch, the diffraction pattern of the second actin-layer corresponding to Tm’s azimuthal positional displacement could be detected and shown to change prior to contraction. Thus, they reported unique troponin-tropomyosin-linked structural changes in the thin filaments during contraction. Interestingly, they further reported evidence of altered Tm displacement in overstretched muscle wherein thin and thick myofilaments no longer overlapped, indicating a role for myosin in thin filament regulation. Following this paradigm, we find here, through tracking the TnC biosensor transient, the time sensitive structural changes in the myofilaments that are essential for regulatory control of the sarcomere.

Overall, the new findings shown here demonstrate a novel FRET-based myofilament activation biosensor valuable for understanding skeletal muscle fiber sarcomere activation mechanisms in health and disease. This new system may also be further refined to investigate other fundamental questions of muscle function. These could include investigations of the effects of mechano-sensing and sarcomere length on the activation of the sarcomere. Studies implementing myofilament acting drugs or with the genetic introduction of mutations could also be envisaged. This system may be developed to include examination of the role of myosin ON/OFF states in sarcomere activation. In this context, there are numerous inherited skeletal myopathies known to disrupt sarcomere performance, leading to a severe decrease in muscle function and in quality of life^55^. These include Nemaline myopathy, Distal arthrogryposis, over 40 Muscular dystrophies, muscle weakness and fatigue^56–60^. The TnC biosensor system may be able to shed light on the mechanisms by which these diseases influence sarcomere function. In addition, this system may facilitate the drug discovery pipeline in developing novel treatments in the form of sarcomere-targeted small molecule therapy. This work also sets the foundation for future studies employing the biosensor to interrogate sarcomere regulation at the level of inter-myofilament signaling, specifically examining the roles of potential muscle mechano-sensors including myosin-binding protein C and titin, as well as numerous other sarcomere-based components in regulating myofilament performance in live skeletal muscles.

## Supplementary Information

Supplementary Figure 1.

Supplementary Figure 2.

## Data Availability

The datasets used and/or analyzed during the current study are available from the corresponding author on reasonable request.
