# Supplementary material for: Sarcomere dynamics revealed by a myofilament integrated FRET-based biosensor in live skeletal muscle fibers
Source: Sci Rep. 2022 Oct 27;12:18116. doi: 10.1038/s41598-022-21425-8 (PMC9613882; doi:10.1038/s41598-022-21425-8)
Supplement: Supplementary file 1 — Supplementary Figure 1. [file 41598_2022_21425_MOESM1_ESM.pdf]

A

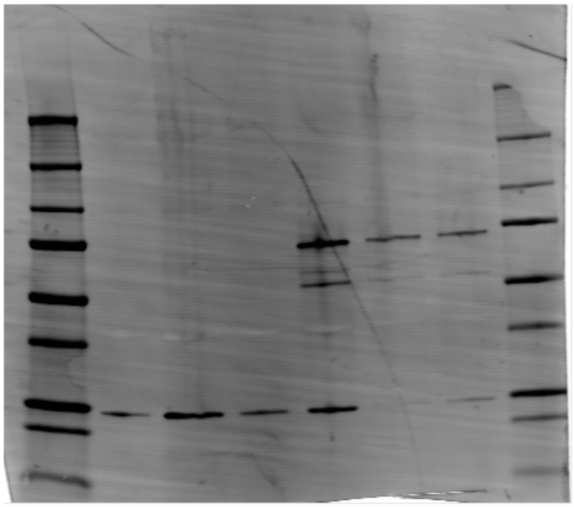

B

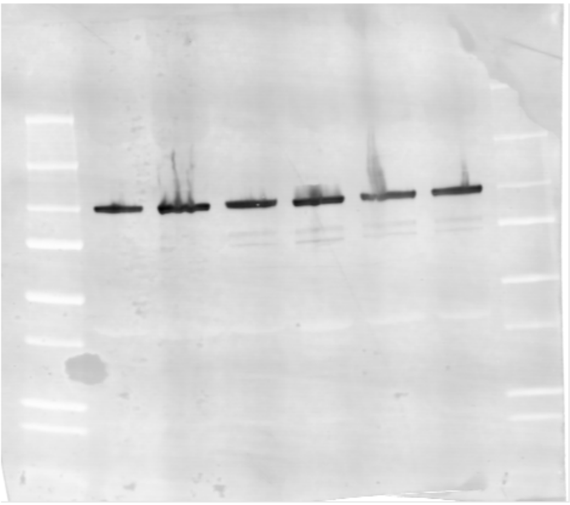

Supplemental Figure 1. Full western blot used for Figure 1D. A. Blot exposure at 700nm (LI-COR Odyssey System) for visualization of fsTnC antibody staining. **B.** Blot exposure at 800nm (LI-COR Odyssey System) for visualization of α-actinin antibody staining.
